# Center frequency as optimal frequency of visual stimulation for spreading entrained gamma rhythms to other target brain regions in cognitively normal older adults

**DOI:** 10.1007/s11357-025-01552-6

**Published:** 2025-02-18

**Authors:** Euisuk Yoon, Yeseung Park, Hong Jun Kim, Jaehyeok Park, Ji Won Han, Se Joon Woo, Seunghyup Yoo, Ki Woong Kim

**Affiliations:** 1 Department of Brain and Cognitive Science, Seoul National University College of Natural Sciences, Seoul, Korea; 2 Department of Neuropsychiatry, Seoul National University Bundang Hospital, 82, Gumi-Ro 173 Beon-Gil, Bundang-Gu, Seongnam-Si, Gyeonggi-Do 13620 Republic of Korea; 3 School of Electrical Engineering, Korea Advanced Institute of Science and Technology (KAIST), Daejeon, Republic of Korea; 4 Department of Ophthalmology, Seoul National University College of Medicine, Seoul, Republic of Korea; 5 Department of Ophthalmology, Seoul National University Bundang Hospital, Seongnam, Republic of Korea; 6 Department of Psychiatry, Seoul National University College of Medicine, Seoul, Korea; 7 Department of Health Science and Technology, Seoul National University Graduate School of Convergence Science and Technology, Suwon, Korea; 8 Institute of Human Behavioral Medicine, Seoul National University Medical Research Center, Seoul, Republic of Korea

**Keywords:** Electroencephalography, Visual evoked potential, Alzheimer disease, Gamma rhythm

## Abstract

Gamma entrainment using 40 Hz sensory stimulation has shown promise in AD mouse models, but inconsistent results in AD patients, possibly due to interspecies and interindividual differences in center frequency (CF). This study aimed to investigate whether gamma rhythms entrained by visual stimulation at an individual’s CF can spread better than those at other frequency conditions in older adults. We entrained gamma rhythms in 32 cognitively normal older participants using light flickering at 32 Hz, 34 Hz, 36 Hz, 38 Hz, and 40 Hz. We identified each individual’s CF among these five frequencies and compared the spread, strength, and stability of gamma connectivity induced by light stimulation flickering at an individual’s CF with those at other frequencies using generalized estimating equation and repeated measures ANOVA. In about two-thirds of the participants, 32 Hz (40.6%) and 34 Hz (28.1%) were identified as CF. The mean spread, strength, and stability of gamma connectivity involving the visual cortex (GC_V-NV_) were higher than those do not involve the visual cortex (GC_NV-NV_, *p* < 0.05). Between the visual cortex and other brain regions, FLS induced with frequencies of high event related spectral perturbation values, including CF and non-center frequency (NCF) 1, generally induced broader, stronger, and more stable gamma connectivity compared to most other NCFs (*p* < 0.001 when comparing NCFs with either CF and NCF1 for both strength and spread; *p* = 0.012 when comparing CF and NCF3 for stability). Gamma rhythms entrained by visual stimulation may better spread to other brain regions when its frequency matched to the individual’s CF.

**Supplementary Information:**

The online version contains supplementary material available at 10.1007/s11357-025-01552-6.

## Introduction

Gamma rhythms, which are associated with many cognitive processes [1, 2], are abnormal in Alzheimer’s disease (AD) [3]. The power of resting gamma rhythms begins to decline early in AD [4], and this decline is closely related to the severity of AD-related pathology [5] and cognitive impairment [6]. Furthermore, the gamma rhythm response to external stimulation not only is slower but also exhibits reduced power and connectivity [3, 7].

Entraining gamma rhythms using 40 Hz visual and/or auditory sensory stimulation has shown promise in reducing amyloid beta (Aβ) accumulation and improving cognitive function in AD mouse models [8]. However, these benefits have not been consistently replicated in AD patients [9, 10]. While there are many possible reasons for this difference in the effectiveness of gamma entrainment between AD mouse models and AD patients, interspecies differences in the optimal frequency of sensory stimulation to induce gamma waves are likely to be one of the key factors driving this difference [11, 12]. The optimal frequency range of frequencies that best entrain gamma rhythms in humans is lower than that in animals including mice [11, 13, 14]. Furthermore, the optimal frequency range for gamma entrainment in older adults was around 32–34 Hz, which was lower than in younger adults, possibly due to the age-associated decline in center frequency (CF) [15].

CF is the frequency at which event-related spectral perturbation (ERSP) shows the most significant change in response to external visual stimulation [13]. Although CF declines with age as contrast sensitivity and excitability of GABAergic inhibitory interneurons decline [16–20], the rate of these changes may not be uniform across individuals and thus CF may vary even within older adults. In addition, although sensory stimulation at the frequency matched to an individual’s CF may entrain gamma rhythms more strongly in the sensory cortices, it is not known whether gamma rhythms entrained by the sensory stimulation at the frequency matched to an individual’s CF are better able to propagate from the sensory cortices to other target brain regions than those entrained by the sensory stimulation at other non-CF frequencies.

This study investigated whether gamma rhythms entrained by visual stimulation flickering at CF are better able to spread from the visual cortex to other brain regions than those entrained by visual situation flickering at other non-CF frequencies by comparing the spread, strength, and stability of gamma connectivity by flickering light stimulation (FLS) in the brain between them. We hypothesized that (1) gamma rhythm connectivity involving the visual cortex (GC_V-NV_) may show greater spread, strength, and stability than those not involved in the visual cortex (GC_NV-NV_) after FLS if FLS-induced gamma rhythms propagate from the visual cortex to other brain regions, and (2) the spread, strength, and stability of GC_V-NV_ induced by FLS at CF (FLS_CF_) may be greater than those induced by FLS at non-CF (FLS_NCF_) if gamma rhythms entrained by FLS_CF_ propagate better than those entrained by FLS_NCF_.

## Methods

### Participants

We enrolled 44 cognitively normal volunteers aged 60 years or older (21 men and 23 women; mean age = 69.9 ± 2.3 years). Geriatric psychiatrists performed standardized diagnostic interviews, physical and neurological examinations, and laboratory tests using the Korean versions of the Consortium to Establish a Registry for Alzheimer’s Disease Assessment Packet [21] and the Mini International Neuropsychiatric Interview [22]. We defined “cognitively normal” as a score greater than above − 1.5 standard deviations based on age-, sex-, and education-adjusted norms for older Korean adults on all neuropsychological tests, and a Clinical Dementia Rating of 0. All participants had normal or corrected-to-normal vision and no hearing impairment. None had a history of major psychiatric or neurological disorders.

Of the 44 participants, 32 were included in the final analysis after exclusion of the following conditions: 3 withdrew consent, 8 had excessive electromyogram (EMG) noise in their electroencephalogram (EEG), and 1 lost EEG digitization data.

### Administration of FLS

This study was conducted as part of our previous work [15, 23]. We delivered the FLS to each participant using a pair of white organic light-emitting diode (OLED) panels (4.7 cm × 4.7 cm; color temperature 3000 K; LG Display Co., Ltd., Seoul, Korea) attached to a pair of eyeglasses. We measured the voltage-luminance characteristics of the OLED panels using a calibrated spectroradiometer (CS2000, Konica-Minolta Inc. Tokyo, Japan) in voltage-controlled mode using a precision source measurement unit (Keithley 2400, Tektronix Inc., Beaverton, OR, USA). We changed the intensity and frequency of the FLS by modulating the amplitude and frequency of the square rhythm using an in-house LabVIEW program (National Instruments Corporation, Austin, TX, USA), which adjusts the intensity of FLS by modulating the supply voltage of the OLED. We used five different flickering frequencies (32 Hz, 34 Hz, 36 Hz, 38 Hz, and 40 Hz).

In this analysis, we only used the ERSP and gamma connectivity induced by 700 cd/m^2^ white FLS of five flickering frequencies (32 Hz, 34 Hz, 36 Hz, 38 Hz, and 40 Hz) because 700 cd/m^2^ white FLS was found to entrain the strongest gamma rhythms in the visual cortex of older adults in our previous work [15, 23].

### MRI acquisition and preprocessing

All participants underwent brain MRI using a Philips Achieva 3 T MR scanner (Philips Medical Systems; Eindhoven, The Netherlands). Three-dimensional T1-weighted MR images were acquired in Digital Imaging and Communications in Medicine (DICOM) format with the following protocol: repetition time/echo time = 8.2 ms/4.6 ms, acquisition matrix = 175 × 240 × 240 resulting in a voxel size of 1.0 × 0.5 × 0.5mm^3^, slice thickness = 1 mm, and flip angle = 8°. Images in DICOM format were then converted to Neuroimaging Informatics Technology Initiative (NIFTI) format using MRIcron software (https://www.nitrc.org/projects/mricron). Images in NIFIT format were resliced into 1 × 1 × 1 mm^3^ isotropic voxels using mri_convert, and the whole brain structures were automatically segmented using recon-all in Freesurfer version 6.0 (https://surfer.nmr.mgh.harvard.edu/).

### Recording and preprocessing of EEG

EEG was recorded with 64 Ag–AgCl electrodes on elastic caps (Easycap, EASYCAP GmbH, Munich, Germany) according to the extended international 10–20 system. FCz was the reference electrode. The forehead was used as the ground electrode, and a pair of electrodes was placed above and below the left eye to record an EMG. The electrode impedance was maintained at 10 kΩ or less throughout the recording. A 24-bit ActiCHamp DC amplifier and a BrainVision Recorder (Brain Products GmbH, Gilching, Germany) amplified and stored the recorded EEG signal. The sampling rate was 1000 Hz. No online filters were applied to the EEG recording. The stimulus markers were delivered by the FLS control system and synchronized with the resting state EEG (rsEEG).

MATLAB (The MathWorks Inc., Natick, MA, USA), EEGLAB [24], and BSMART [21] toolboxes were used for preprocessing and analysis. We filtered the recorded signals with a 1-Hz high-pass finite impulse response filter and a 60-Hz notch filter and then applied them to a common average reference. We performed independent component analysis to remove eye blinks and other ocular artifacts from the EEG signal. After preprocessing, we segmented 5-min rsEEG recordings into 500-ms epochs and then randomly selected 20 artifact-free epochs from them. We obtained a 4000-ms epoch from 1000 ms before each FLS onset to 1000 ms after each FLS offset, resulting in twenty 4000-ms epochs of each frequency. Each 4000 ms EEG data acquired during FLS was then divided into 500 ms time windows with 250 ms overlap, resulting in 15 consecutive overlapping data segments. Among them, 7 data segments from the 5th to the 12th segment represent 2000 ms EEG signal during FLS.

### Identification of Individual CF

In each participant, individual CF was identified using FLS-induced ERSP of four channels (Oz, POz, O1, and O2) [13, 25]. The ERSP was calculated using the newtimef function, as provided by EEGLAB [24]. The recorded EEG data was divided into epochs of 4 s in duration, comprising 1 s prior to the onset of stimulation, 2 s during stimulation, and 1 s following the offset of stimulation. The baseline period employed for normalization was the 1000 ms pre-onset of FLS. ERSP values were calculated with difference in ERS during the 2-s FLS stimulation period from the ERS during the 1000-ms pre-onset of FLS at each flickering frequency, resulting in five distinct ERSP values (32, 34, 36, 38, and 40 Hz) per subject. The details of the ERSP estimation are described in our previous work [15, 23]. The five flickering frequencies (32 Hz, 34 Hz, 36 Hz, 38 Hz, and 40 Hz) at each channel were ranked based on ERSP, and an average rank of the four channels at each flickering frequency was obtained. The flickering frequency with the highest mean rank was defined as CF. In cases where two or more frequencies had the same mean rank, the flickering frequency with the highest absolute ERSP in any of the four channels was defined as CF. The other four flickering frequencies were defined as NCF1, NCF2, NCF3, and NCF4 in the order of ERSP. The supplementary figure was drawn to show the proportions of different frequency distances to CF for each of NCF1-4 (Supplementary Fig. 1).

### Analysis of functional gamma connectivity

EEG functional connectivity was measured using the MNE library in Python (https://mne.tools/stable/index.html) [26]. First, the brain surface was extracted from individual T1-weighted images using Freesurfer. Then, the three-layer boundary element model (BEM) [27] with default conductivity values by MNE (0.3, 0.006, and 0.3 for scalp, skull, and brain, respectively) was computed and the source space was generated. We used dynamic statistical parametric mapping (dSPM) for inverse solution [28]. Electrode positions were digitized using 3D model images and manually positioned using EEGLAB. We performed co-registration between the EEG digitization and the individual source space, followed by computation of the forward model and inverse solution.

Using images from the Human Connectome Project [29], 44 different cortices, 22 in each hemisphere, were labeled. The visual region consisted of the ventral stream visual cortex, primary visual cortex, early visual cortex, and dorsal stream visual cortex, resulting in 8 cortices including left and right hemispheres. These were categorized based on their relationship to the corresponding region [29]. Cortices that appeared to overlap with multiple regions were excluded from the visual regions, and connectivity between visual cortices was also excluded as our focus is to find gamma connectivity from visual to other regions, or the effect of gamma propagation by FLS. Regional gamma connectivity was assessed using the phase locking value (PLV) [30] in both rsEEG and EEG acquired during FLS. The frequency range for measuring PLV is ± 1 of the frequency of FLS. In the PLV matrix, the total number of edges of all possible connectivity is 1892. Of these, 576 edges were included in the GC_V-NV_ and 1000 edges in the GC_NV-NV_. GC_V-NV_ was measured to evaluate the gamma connectivity induced by gamma rhythm FLS, while GC_NV-NV_ was used as a negative control to compare with GC_V-NV_ and demonstrate that FLS gamma entrainment was properly propagated by visual stimulation and does not represent intrinsic gamma rhythm.

To identify the gamma connectivity induced by FLS, the PLV of the seven data segments of each edge during FLS was compared with that in rsEEG using paired t-tests with Green-Geisser non-sphericity correction and Bonferroni post hoc comparisons. The edges that showed significantly higher PLV in any of the seven data segments during FLS compared to that in rsEEG were defined as E_FLS_ which indicates FLS-induced gamma connectivity. Among E_FLS_, those showing significantly stronger PLV than rsEEG for more than 3 data segments during FLS were defined as sE_FLS_ which indicates FLS-induced stable gamma connectivity. Then, the spread, stability, and strength of E_FLS_ at each frequency condition were calculated to measure how wide the increase in gamma connectivity is, how long the increased gamma connectivity is maintained uninterrupted, and how strong the increase in gamma connectivity is, respectively. Spread was defined as the proportion (%) of E_FLS_ among all possible edges. Stability was defined as the proportion (%) of sE_FLS_ among all E_FLS_. Strength was defined as the mean PLV of the seven data segments during FLS of all E_FLS_. These mentioned measurement methods were both done across CF-NCFs and specific frequencies (32 Hz, 34 Hz, 36 Hz, 38 Hz, 40 Hz).

To provide better insight into gamma connectivity by FLS, we have added PLV over time across CF-NCFs and specific frequencies for both GC_V-NV_ and GC_NV-NV_ (Supplementary Fig. 2). Each time series shows a gray region representing the baseline PLV values, which averaged 250 ms of 20 epochs in resting state, while the period from 1 to 2000 ms shows PLV values, which averaged 2000 ms of 20 epochs during FLS. Additionally, we have visualized PLV-based connectivity using 3D brain plots generated with MATLAB using ICBM template given by the brainstorm [31]. The 3D plots show source localization averaged across all subjects, highlighting PLV connections during FLS that both showed significant differences from resting state and exceeded the conventional threshold of 0.7 [32, 33]. GC_V-NV_ connections are depicted in red and GC_NV-NV_ connections in blue (Supplementary Fig. 3).

### Statistical analyses

Continuous and categorical variables were compared between groups using Student *t* test and chi-square test, respectively.

GEE analysis and multinomial logistic regression analysis were used to evaluate whether there were differences in specific frequency (32, 34, 36, 38, and 40 Hz) distributions according to each CF and NCFs. Subsequently, multinomial logistic regression analysis was used to evaluate how often specific frequencies appeared relatively in each case, using CF as a reference.

The effects of regions of gamma connectivity (GC_V-NV_ and GC_NV-NV_), flickering frequencies classified by ERSP (CF, NCF1, NCF2, NCF3, and NCF4), and specific frequencies (32 Hz, 34 Hz, 36 Hz, 38 Hz, 40 Hz) of entrained gamma rhythms in the visual cortex, as well as their interaction on the induction of E_FLS_ (spread) and sE_FLS_ (stability) were examined using generalized estimating equation (GEE) with Bonferroni post hoc comparisons. The effects of regions of gamma connectivity (GC_V-NV_ and GC_NV-NV_), flickering frequencies classified by ERSP (CF, NCF1, NCF2, NCF3, and NCF4), and specific frequencies (32 Hz, 34 Hz, 36 Hz, 38 Hz, 40 Hz) of entrained gamma rhythms in the visual cortex, as well as their interaction on the strength of E_FLS_ were examined using rmANOVA with Bonferroni post hoc comparisons.

All analyses were performed using SPSS for Windows (version 20.0; IBM Co., Armonk, NY, USA) and MATLAB (The MathWorks Inc., Natick, MA, USA).

## Results

**Fig. 1:**
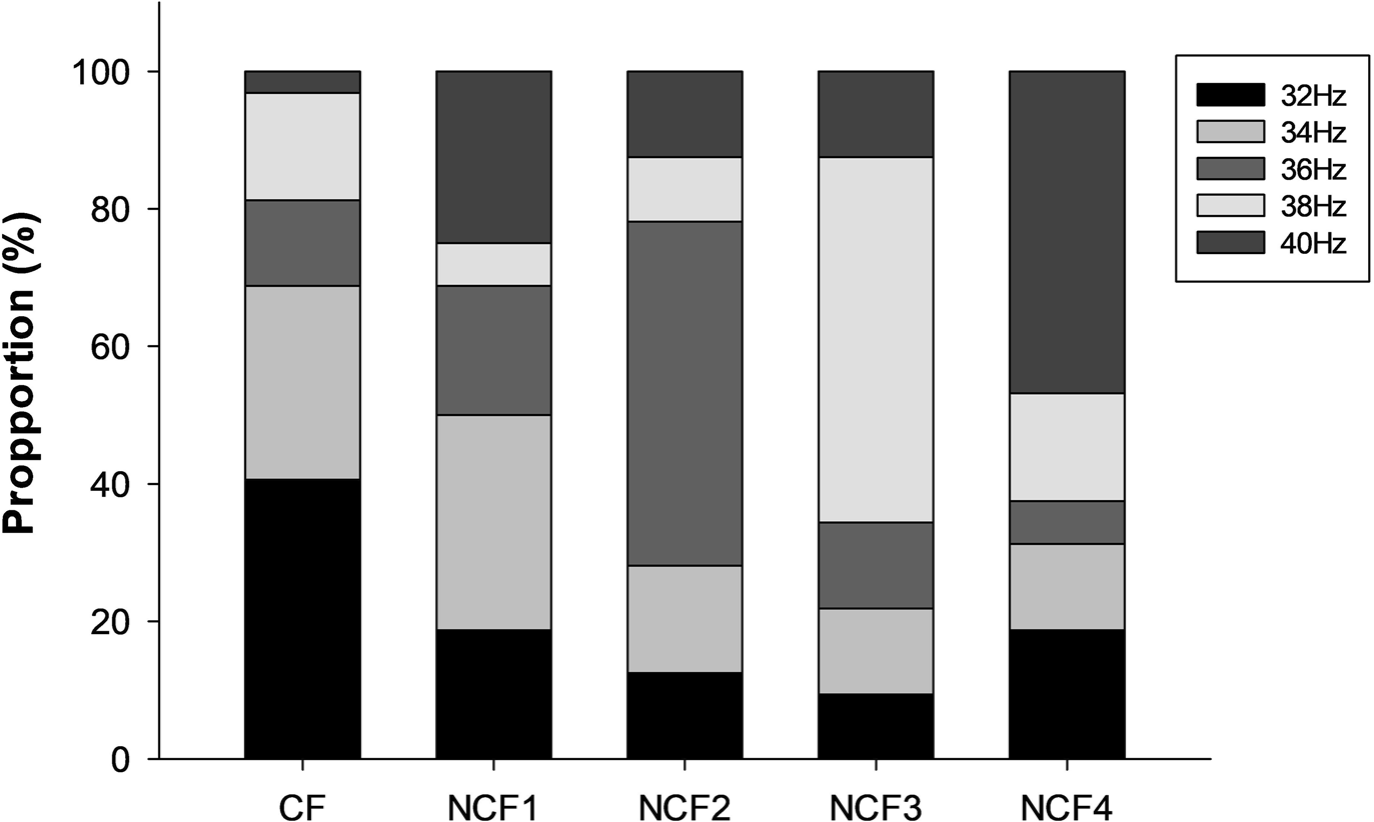
Distribution of flickering frequencies between center and non-center frequencies. CF, center frequency; NCF1, non-center frequency with the second highest event-related spectral perturbation (ERSP) of flickering light stimulation (FLS)–entrained gamma rhythms; NCF2, non-center frequency with the third highest ERSP of FLS-entrained gamma rhythms; NCF3, non-center frequency with the fourth highest ERSP of FLS-entrained gamma rhythms; NCF4, non-center frequency with the lowest ERSP of FLS-entrained gamma rhythms

Demographic and clinical characteristics were comparable between included and excluded participants (Table 1), and distribution of specific frequencies on center frequency is drawn (Fig. 1).

**Table 1:** Characteristics of the participants

	Included (*n* = 32)	Excluded (*n* = 12)	p*
Age, years, mean (SD)	69.9 (2.4)	69.4 (1.9)	0.525
Sex, women, *n* (%)	17 (53.1)	6 (50.0)	1.000
Education, years, mean (SD)	11.6 (4.6)	13.8 (2.2)	0.126
MMSE, points, mean (SD)	28.2 (2.0)	28.2 (1.6)	0.937
GDS, points, mean (SD)	7.2 (5.2)	8.1 (6.6)	0.619

*SD* standard deviation, *MMSE* Mini Mental Status Exam, *GDS* Geriatric Depression Scale

*Students *t*-test for continuous variables and chi-square test for categorical variable

The GEE model showed that specific frequencies had a significant impact on CF and NCF (*W* = 19.349, *p* < 0.001), which was statistically significant. This suggests that specific frequencies significantly influence center frequencies. Subsequently, analysis for multinomial logistic regression showed that in NCF2, the probability of frequency 36 Hz appearing was about 5.33 times higher compared to CF, which showed a statistically significant difference (Exp(*B*) = 5.333, *p* < 0.01). On the other hand, in NCF3, the probabilities of frequencies 32 Hz and 38 Hz appearing were approximately 0.17 times (Exp(*B*) = 0.167, *p* < 0.01) lower and 3 times (Exp(*B*) = 3.000, *p* < 0.05) higher compared to CF, respectively, which were statistically significant. Additionally, in NCF4, the probability of frequency 40 Hz appearing was six times higher (Exp(*B*) = 6, *p* = 0.019) compared to CF. In CF, lower frequency was more preferred, while the probability for higher frequency decreased. These results indicate that the specific frequency can vary depending on CF and NCF.

**Fig. 2:**
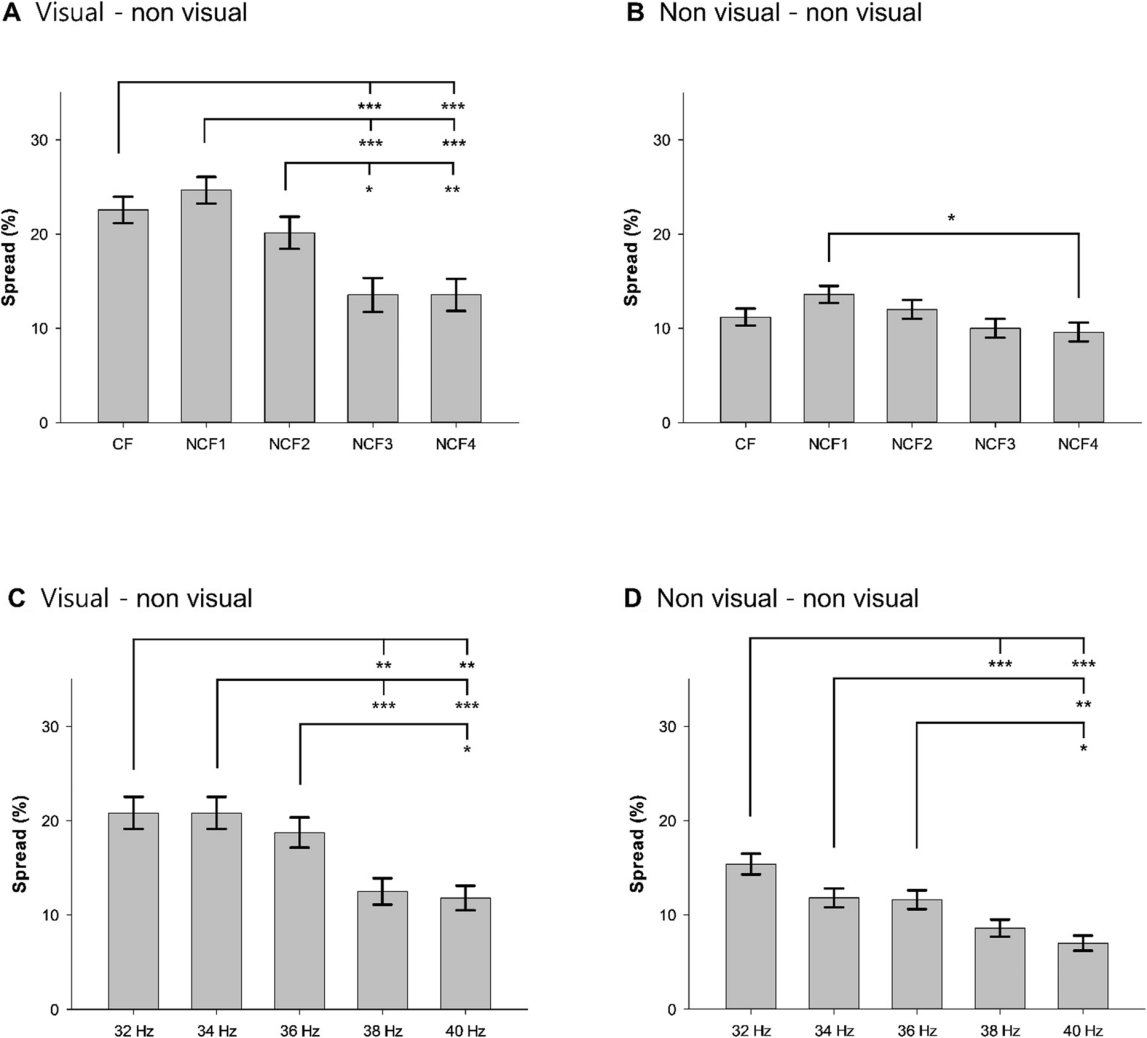
Spread of gamma connectivity during flickering light stimulation. **A** Gamma connectivity defined by CF involving the visual cortex. **B** Gamma connectivity defined by CF not involving the visual cortex. **C** Gamma connectivity defined by specific frequencies involving the visual cortex. **D** Gamma connectivity defined by specific frequencies not involving the visual cortex. CF, center frequency; NCF1, non-center frequency with the second highest event-related spectral perturbation (ERSP) of flickering light stimulation (FLS)–entrained gamma rhythms; NCF2, non-center frequency with the third highest ERSP of FLS-entrained gamma rhythms; NCF3, non-center frequency with the fourth highest ERSP of FLS-entrained gamma rhythms; NCF4, non-center frequency with the lowest ERSP of FLS-entrained gamma rhythms; Error bars indicate standard errors. *p < 0.05, **p < 0.01, ***p < 0.001 by generalized estimating equation with Bonferroni post hoc comparisons

As summarized in Table 2, the main effects of both the region of gamma connectivity (GC_V-NV_ and GC_NV-NV_) and the flickering frequency classified by ERSP (CF, NCF1, NCF2, NCF3, and NCF4) on the spread and stability of gamma connectivity were significant in the GEE model (*W* = 62.548, *p* < 0.001 for region of gamma connectivity; *W* = 52.339, *p* < 0.001 for flickering frequency). Their interaction between them showed marginal statistical significance (*W* = 9.444, *p* = 0.051). The mean spread of GC_V-NV_ (18.9 ± 39.1%) was about 1.6 times higher than that of GC_V-NV_ (10.8 ± 31.1%). When GC_V-NV_ and GC_V-NV_ were analyzed separately, the spread was significantly different between CF and NCFs in both GC_V-NV_ (*W* = 620.237, *p* < 0.001) and GC_NV-NV_ (*W* = 1617.402, *p* = 0.016). In GC_V-NV_, the spread induced by FLS_CF_ and FLS_NCF1_ was significantly higher than that induced by FLS_NCF3_ and FLS_NCF4_ (*p* < 0.001). However, in GC_NV-NV_, the spread induced by FLS_CF_ was comparable to that induced by FLS in NCFs (*p* > 0.05). These results suggest that gamma rhythms entrained in the visual cortex by FLS in CF or NCF1 may be more widespread than those entrained by FLS in other NCFs (Fig. 2A, B).

**Table 2:** Effects of region of gamma connectivity and flickering frequency classified by event-related spectral perturbation (ERSP) of entrained gamma rhythms on the spread, strength, and stability of gamma connectivity

	FF							RGC				FF × RGC	
	CF	NCF1	NCF2	NCF3	NCF4	W/F^**a**^	*p*^**a**^	V-NV	NV-NV	W/F^**a**^	*p*^**a**^	W/F^**a**^	*p*^**a**^
Spread, %, mean (SE)^a^	15.7 (0.9)	18.1 (1)	15.3 (0.9)	11.4 (0.8)	11.2 (0.8)	52.349	< 0.001	18.4 (0.9)	10.7 (0.5)	62.548	< 0.001	9.444	0.051
Strength, mean (SE)	0.19 (0.03)	0.24 (0.04)	0.22 (0.04)	0.15 (0.04)	0.16 (0.04)	234.399	< 0.001	0.22 (0.03)	0.19 (0.03)	99.320	< 0.001	84.718	< 0.001
Stability, %, mean (SE)^a^	24.8 (2.8)	22.2 (2.5)	15.2 (2.3)	11.3 (2.4)	20.6 (3.1)	15.476	0.004	21.8 (1.9)	15.2 (1.6)	7.158	0.007	2.296	0.681

*FF* flickering frequency, *RGC* region of gamma connectivity, *CF* center frequency, *NCF1* non-center frequency with the second highest event-related spectral perturbation (ERSP) of flickering light stimulation (FLS)–entrained gamma rhythms, *NCF2* non-center frequency with the third highest ERSP of FLS-entrained gamma rhythms, *NCF3* non-center frequency with the fourth highest ERSP of FLS-entrained gamma rhythms, *NCF4* non-center frequency with the lowest ERSP of FLS-entrained gamma rhythms, *V-NV* gamma connectivity between the visual cortex and other cortices, *NV-NV* gamma connectivity between cortices other than the visual cortex, *SE* standard error

^**a**^Generalized estimating equation for spread and stability and repeated measures analysis of variance for strength

The main effects of both the region of gamma connectivity (GC_V-NV_ and GC_NV-NV_) and the specific flickering frequency (32 Hz, 34 Hz, 36 Hz, 38 Hz, 40 Hz) on the spread and stability of gamma connectivity were significant in the GEE model (*W* = 45.08, *p* < 0.001 for region of gamma connectivity; *W* = 71.088, *p* < 0.001 for flickering frequency). Their interaction between them showed no statistical significance (*W* = 3.437, *p* = 0.487). The mean spread of GC_V-NV_ (16.9 ± 37.5%) was about 1.6 times higher than that of GC_V-NV_ (10.4 ± 30.6%). When GC_V-NV_ and GC_V-NV_ were analyzed separately, the spread was significantly different between CF and NCFs in both GC_V-NV_ (*W* = 629.756, *p* < 0.001) and GC_NV-NV_ (*W* = 1611.754, *p* < 0.001). In GC_V-NV_, the spread induced by FLS_32Hz_, FLS_34Hz_, and FLS_36Hz_ was significantly higher than that induced by FLS_38Hz_ and FLS_40Hz_ (*p* < 0.001). In GC_NV-NV_, the spread induced by FLS_32Hz_ was significantly higher than FLS_38Hz_ and FLS_40Hz_ (*p* < 0.001), and FLS_34Hz_ was significantly higher than that induced by FLS_40Hz_ (*p* < 0.01). These results suggest that gamma rhythms entrained in the visual cortex by FLS in lower frequencies (32 and 34 Hz) may be more widespread than those entrained by FLS in higher frequencies (Fig. 2C, D).

**Fig. 3:**
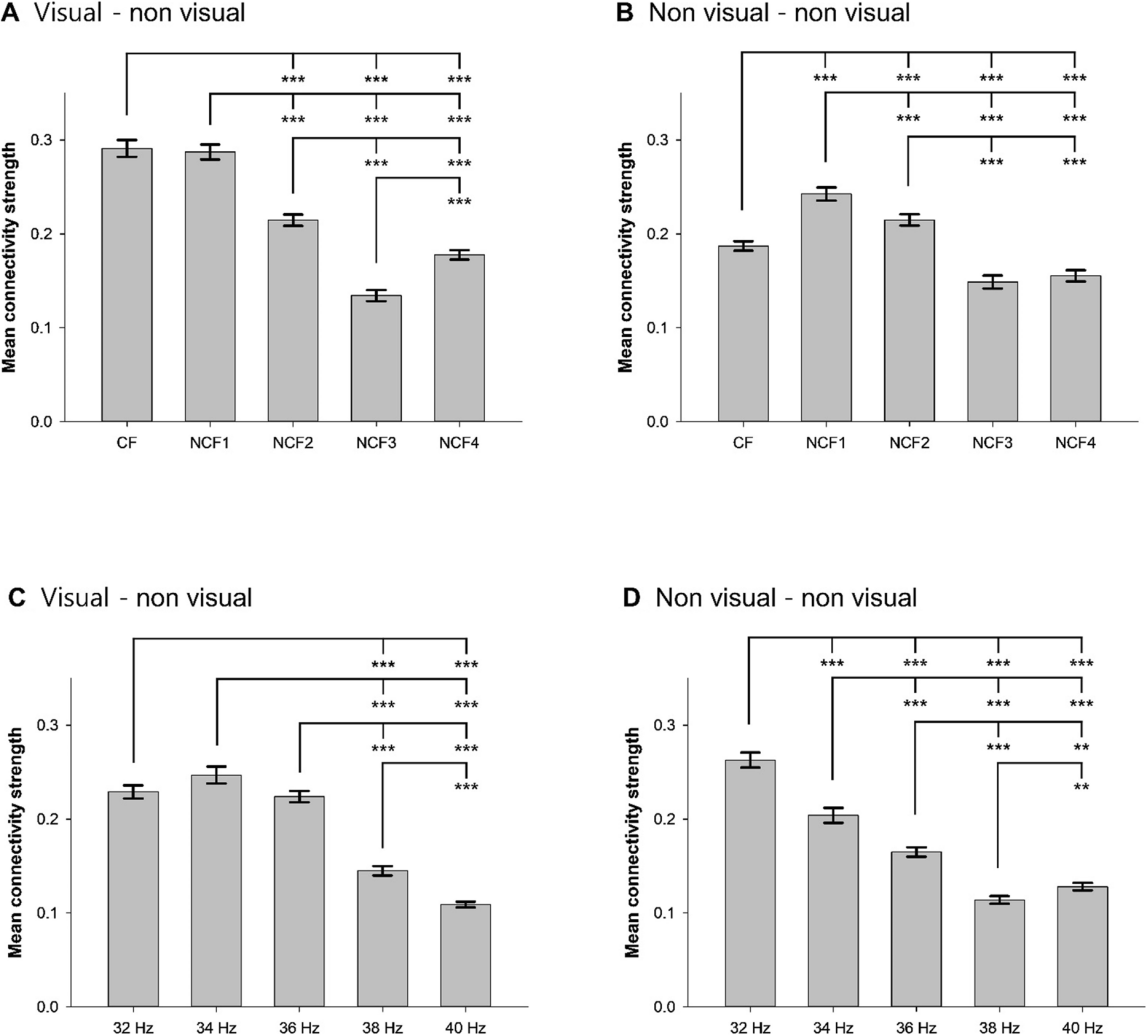
Strength of gamma connectivity during flickering light stimulation. **A** Gamma connectivity defined by CF involving the visual cortex. **B** Gamma connectivity defined by CF not involving the visual cortex. **C** Gamma connectivity defined by specific frequencies involving the visual cortex. **D** Gamma connectivity defined by specific frequencies not involving the visual cortex. CF, center frequency; NCF1, non-center frequency with the second highest event-related spectral perturbation (ERSP) of flickering light stimulation (FLS)–entrained gamma rhythms; NCF2, non-center frequency with the third highest ERSP of FLS-entrained gamma rhythms; NCF3, non-center frequency with the fourth highest ERSP of FLS-entrained gamma rhythms; NCF4, non-center frequency with the lowest ERSP of FLS-entrained gamma rhythms; Error bars indicate standard errors. **p < 0.01, ***p < 0.001 by repeated measures analysis of variance with Bonferroni post hoc comparisons

The main effects of both region of gamma connectivity, flickering frequency classified by ERSP, and their interaction on the strength of gamma connectivity were all significant in the rmANOVA (*F*_1,1_ = 99.320, *p* < 0.001 for region of gamma connectivity; *F*_1,4_ = 234.399, *p* < 0.001 for flickering frequency; *F*_1,4_ = 84.718, *p* < 0.001 for their interaction; Table 2). The mean strength of GC_V-NV_ (0.22 ± 0.03) was about 1.15 times higher than that of GC_NV-NV_ (0.19 ± 0.02). When GC_V-NV_ and GC_NV-NV_ were analyzed separately, the strength induced by FLS was significantly different between flickering frequencies in both GC_V-NV_ (*F*_4, 124_ = 241.221, *p* < 0.001) and GC_NV-NV_ (*F*_4, 124_ = 109.125, *p* < 0.001). In the post hoc comparisons, the strength induced by FLS_CF_ was significantly higher than that induced by FLS_NCF2_, FLS_NCF3_, and FLS_NCF4_ (*p* < 0.001) in GC_V-NV_, while significantly higher than that induced by FLS_NCF3_ and FLS_NCF4_ but significantly lower than that induced by FLS_NCF1_ and FLS_NCF2_ in GC_NV-NV_ (*p* < 0.001). These results suggest that gamma rhythms in other brain regions may be more tightly phase-locked to those entrained by the FLS in the visual cortex when flickering frequency of the FLS is matched to the individual CF than when it is not (Fig. 3A, B).

The main effects of both region of gamma connectivity, specific flickering frequency, and their interaction on the strength of gamma connectivity were all significant in the rmANOVA (*F*_1,1_ = 31.838, *p* < 0.001 for region of gamma connectivity; *F*_1,4_ = 196.662, *p* < 0.001 for flickering frequency; *F*_1,4_ = 172.892, *p* < 0.001 for their interaction). The mean strength of GC_V-NV_ (0.22 ± 0.03) was about 1.15 times higher than that of GC_NV-NV_ (0.19 ± 0.03). When GC_V-NV_ and GC_NV-NV_ were analyzed separately, the strength induced by FLS was significantly different between flickering frequencies in both GC_V-NV_ (*F*_4, 124_ = 220.677, *p* < 0.001) and GC_NV-NV_ (*F*_4, 124_ = 154.660, *p* < 0.001). In the post hoc comparisons, the strength induced by FLS_32Hz_ and FLS_34Hz_ were significantly higher than that induced by FLS_38Hz_ and FLS_40Hz_ (*p* < 0.001) in GC_V-NV_, while FLS_32Hz_ was significantly higher than that induced by FLS_34Hz_, FLS_36Hz_, FLS_38Hz_, and FLS_40Hz_ in GC_NV-NV_ (*p* < 0.001). These results suggest that gamma rhythms in other brain regions may be more tightly phase-locked to those entrained by the FLS in the visual cortex when flickering frequency of the FLS is lower (Fig. 3C, D).

**Fig. 4:**
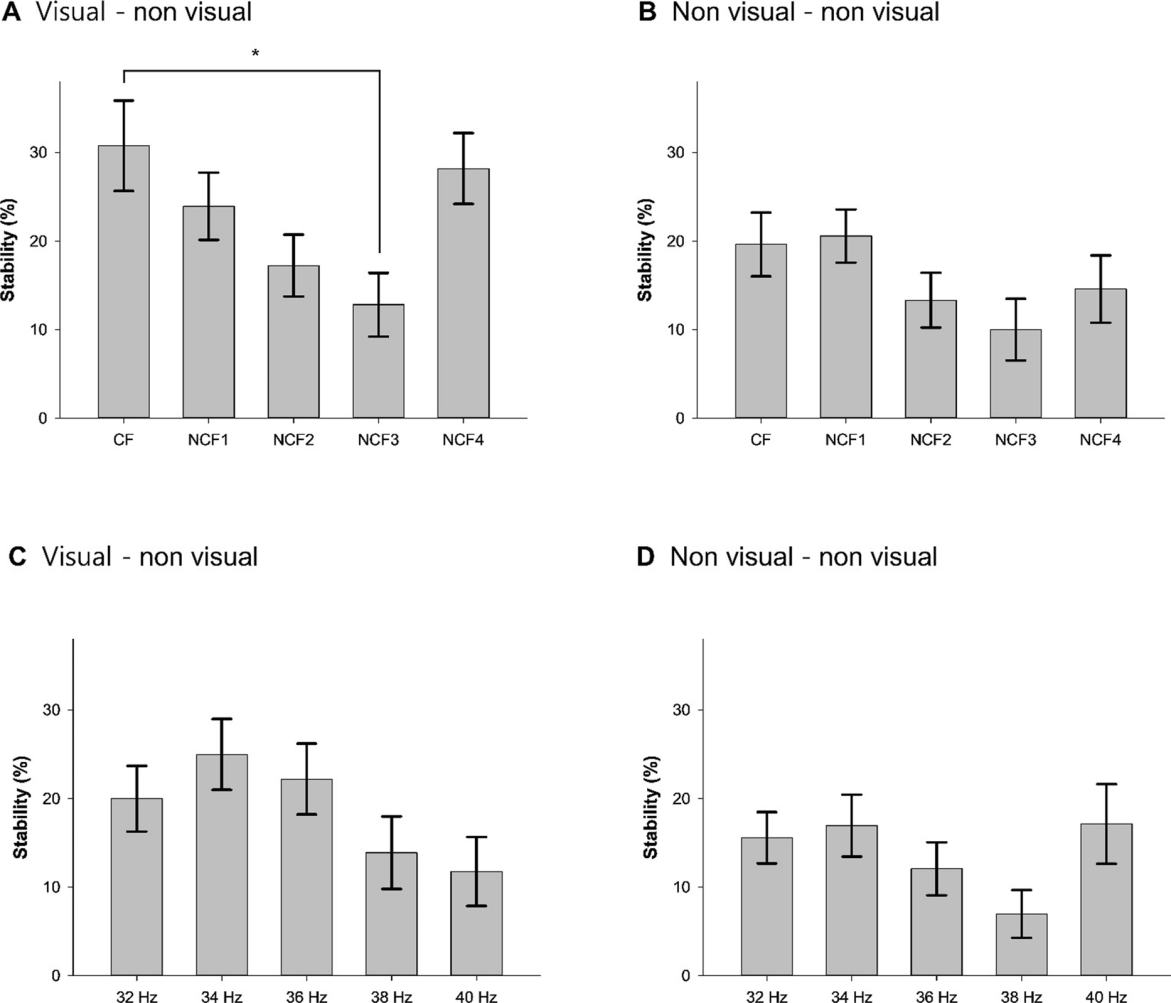
Stability of gamma connectivity during flickering light stimulation. **A** Gamma connectivity defined by CF involving the visual cortex. **B** Gamma connectivity defined by CF not involving the visual cortex. **C** Gamma connectivity defined by specific frequencies involving the visual cortex. **D** Gamma connectivity defined by specific frequencies not involving the visual cortex. CF, center frequency; NCF1, non-center frequency with the second highest event-related spectral perturbation (ERSP) of flickering light stimulation (FLS)–entrained gamma rhythms; NCF2, non-center frequency with the third highest ERSP of FLS-entrained gamma rhythms; NCF3, non-center frequency with the fourth highest ERSP of FLS-entrained gamma rhythms; NCF4, non-center frequency with the lowest ERSP of FLS-entrained gamma rhythms; error bars indicate standard errors. *p < 0.05 by generalized estimating equation with Bonferroni post hoc comparisons

The main effects of region of gamma connectivity and flickering frequency classified by ERSP on the stability of gamma connectivity were also significant in the GEE model (*W* = 7.518, *p* = 0.007 for region of gamma connectivity; *W* = 15.476, *p* = 0.004 for flickering frequency). However, their interaction was not statistically significant (*W* = 2.296, *p* = 0.681; Table 2). The mean stability of GC_V-NV_ (23.2 ± 42.2%) was about 1.5 times higher than that of GC_NV-NV_ (16.0 ± 37.7%). As shown in Fig. 4A, B, when the frequency of FLS was CF, the stability was highest in GC_V-NV_ (40 sE_FLS_ [30.8%] of 130 GC_V-NV_ E_FLS_) and second highest in GC_NV-NV_ (22 sE_FLS_ [19.6%] of 112 GC_NV-NV_ sE_FLS_**)**. When GC_V-NV_ and GC_V-NV_ were analyzed separately, the stability was significantly different between FLS_CF_ and FLS_NCF_ in GC_V-NV_ (*W* = 11.938, *p* = 0.018), and stability induced by FLS_CF_ was significantly higher than that induced by FLS_NCF3_ in GC_V-NV_ in post hoc comparisons (*p* = 0.012). However, in GC_NV-NV_, the stability induced by FLS did not differ between flickering frequencies (*W* = 6.542, *p* = 0.162). These results suggest that gamma rhythms entrained in the visual cortex by FLS_CF_ may spread to other brain regions for a longer duration than those entrained by FLS_NCF_ (Fig. 4A, B).

The main effects of the region of gamma connectivity and specific frequency on the stability of gamma connectivity were also significant in the GEE model (*W* = 3.910, *p* = 0.048). However, their specific flickering on the stability of gamma connectivity and their interaction were not statistically significant (*W* = 8.296, *p* = 0.081 for flickering frequency; *W* = 4.355, *p* = 0.360 for interaction). The mean stability of GC_V-NV_ (19.7 ± 39.8%) was about 1.4 times higher than that of GC_NV-NV_ (14.0 ± 34.7%). When GC_V-NV_ and GC_V-NV_ were analyzed separately, the stability showed no significance between flickering frequencies in GC_V-NV_ (*W* = 7.985, *p* = 0.092). Likewise, in GC_NV-NV_, the stability induced by FLS did not differ between flickering frequencies (*W* = 7.829, *p* = 0.098). These results suggest that gamma rhythms entrained in the visual cortex by lower-frequency FLS may spread to other brain regions for a longer duration than those entrained by higher-frequency FLS (Fig. 4C, D).

## Discussion

This study found that the CF differed among the older participants and that FLS flickering at an individual’s CF increased gamma connectivity between the visual cortex and other brain regions more broadly, more strongly, and more stably than FLS flickering at other frequencies, suggesting that the flickering frequency of FLS for gamma entrainment as an intervention for AD patients’ needs to be individualized according to the individual’s CF.

To date, five clinical trials with small groups of AD patients have investigated the efficacy of gamma entrainment using daily 40 Hz visual or combined visual-auditory stimulation for 1 to 6 months [9, 10, 34–36]. Benefits such as delayed brain atrophy, enhanced network connectivity, and improved cognitive or daily living functions were found in four studies [10, 34–36]. However, Aβ reduction was not assessed in three trials [34–36] and was not observed in two trials [9, 10]. These findings highlight the need for further research to fine-tune stimulation parameters for effective gamma entrainment in AD patients.

This study distinguished frequencies based on ERSP and specific frequencies. We evaluated specific frequency distributions according to each CF and NCFs to both show the relation of specific frequencies and center frequencies. The results and Fig. 1 suggested that while specific frequencies were distributed across the center frequencies, CF has a distribution concentrated in relatively lower frequencies with limited possibility of including high-frequency bands. These differences in frequency distribution show the difference between central frequencies and specific frequencies and may reflect functional differences in neural networks between CF and NCF.

We measured the phase synchrony of gamma rhythms in two different regions using PLV. A higher PLV represents stronger synchronization between two EEG signals [30]. In the current study, gamma connectivity increased significantly after FLS, and the increase was broader, stronger, and more stable in GC_V-NV_ compared to GC_NV-NV_. The greater increase in GC_V-NV_ compared to GC_NV-NV_ may reflect, at least in part, that propagation of gamma rhythms entrained in the visual cortex to other brain regions may contribute to the increase in GC_V-NV_. The current study also showed that, in GC_V-NV_, the FLS-induced increase was broader and stronger when the FLS flickered at individual’s CF than at the other NCF frequencies. NCF1, however, does show some comparable strength and broadness with CF. This can be attributed to NCF1’s higher proportion of closer frequency (2 Hz distance, 75%; 4 Hz distance, 18.75%; 6 Hz distance, 6.25%) with CF (Supplementary Fig. 1). This result may suggest the wider range (from 2 to 4 Hz) of individual CF. This figure also shows higher numbered NCFs have a lower proportion of 2 Hz distance from CF. Thus, this may also suggest that larger frequency differences from individual CFs in NCFs are attributed to the difference in the gamma connectivity. In AD, both the power and functional connectivity of gamma rhythms across multiple brain regions are reduced [37, 38]. Additionally, memory is related to functional gamma connectivity [39, 40]. In mice, cholinesterase inhibitors increased the power and functional connectivity of cortical gamma rhythms [41]. Therefore, stronger and broader entrainment of gamma rhythms may be more effective as a therapeutic intervention of AD. This suggests that adapting the flickering frequency of the FLS to the individual’s CF may be necessary to achieve stronger entrainment and broader spread of gamma rhythms to increase the efficacy of gamma entrainment in AD patients.

We compared the spread, strength, and stability of gamma rhythm connectivity within specific frequencies (Figs. 2, 3, and 4C, D). Similar to the results from CF, the spread and strength of gamma connectivity favored lower gamma rhythms of 32 and 34 Hz. This supports previous studies showing that lower gamma rhythms are favored over higher frequencies [15, 23], which aligns with our finding that CF is mostly comprised of lower frequencies. However, it should be noted that the strength of gamma connectivity was comparable between 32 and 36 Hz, unlike CF and NCF2, which showed significant differences. In addition, the stability for 32 Hz was only the third highest, and while 34 Hz showed the highest stability, it was comparable to other specific frequencies. Hence, we conclude that CF is more reliable in forming wider, stronger, and more stable gamma rhythm connectivity compared to lower gamma rhythms of 32 or 34 Hz.

Sensitivity to visual stimuli differs markedly between diurnal humans and nocturnal mice [12, 42], and optimal range of flickering frequencies that best entrain gamma rhythms in humans (30–40 Hz) is also about 5 Hz lower than in mice (35–45 Hz) [11, 14]. Even in humans, the CF of gamma rhythms decreases by 0.16 Hz per year in the high gamma range (36 Hz or higher) and by 0.08 Hz per year in the low gamma range (below 36 Hz) [25]. CF positively correlates with GABA levels in the visual cortex [43] and declines as the excitability of GABAergic inhibitory interneurons declines [20]. GABA levels are known to decline gradually with age in several brain regions, including the visual and prefrontal cortices [18, 19]. However, the rate of age-associated changes in the GABAergic system, and thus the rate of age-associated decline in CF, may not be uniform among older adults. In the current study, about two thirds of the older participants had CF of 32 Hz or 34 Hz, but still one third of them had CF of 36–40 Hz (Fig. 1). Therefore, CF needs to be identified on an individual basis.

This study has several limitations. First, FLS_CF_ increased gamma connectivity in about 20% of all possible edges between the visual cortex and other brain regions and their mean PLV was about 0.3 which is similar to the resting state PLV of global gamma connectivity in healthy adults [44]. Further research is needed to determine whether this level of gamma entrainment and propagation is broad and strong enough to produce therapeutic effects in AD patients, and if not, what level of gamma entrainment and propagation should be induced. Second, the participants of the current study were all healthy older adults. However, in AD patients, light-scattering of lens increases due to Aβ microaggregates in the lens [45]. Therefore, the FLS parameters including flickering frequency optimal for gamma entrainment in AD patients may be different from those in healthy older adults. Third, the lower limit of the flickering frequency of FLS in the current study was 32 Hz. However, it is possible that the CF may be lower than 32 Hz in some people, especially those with AD.

Despite these limitations, the current study successfully demonstrated for the first time that gamma rhythms entrained by FLS may better spread to other brain regions when the frequency of FLS matched to the individual’s CF.

## Conclusions

This study sheds light on the optimal conditions for FLS that effectively induce gamma functional connectivity in individual humans. Prior FLS studies have focused on specific gamma frequencies to generate robust entrainment. While the general range of 32–38 Hz proves effective in inducing gamma rhythm connectivity, tailoring FLS based on the CF to accommodate individual differences provides advantages beyond strong entrainment alone. Our findings suggest that CF does differ between individuals, and FLS_CF_ prompts significant changes in regions interconnected with visual areas. Moreover, it implies that FLS_CF_ not only results in robust gamma rhythm entrainment but also in wider, stronger, and more stable synchronization across the entire brain during the stimulation period. Therefore, future investigations should consider employing CF to enhance functional connectivity during FLS, with a focus on further improving the therapeutic effectiveness of FLS interventions for AD.

## Supplementary Information

Below is the link to the electronic supplementary material.

Supplementary file1 (DOCX 477 KB)

## Data Availability

The data that support the findings of this study are available on request from the corresponding author.
