# Supplementary material for: Center frequency as optimal frequency of visual stimulation for spreading entrained gamma rhythms to other target brain regions in cognitively normal older adults
Source: GeroScience. 2025 Feb 18;47(3):4451–64. doi: 10.1007/s11357-025-01552-6 (PMC12181565; doi:10.1007/s11357-025-01552-6)
Supplement: Supplementary file 1 — Supplementary file1 (DOCX 477 KB) [file 11357_2025_1552_MOESM1_ESM.docx]

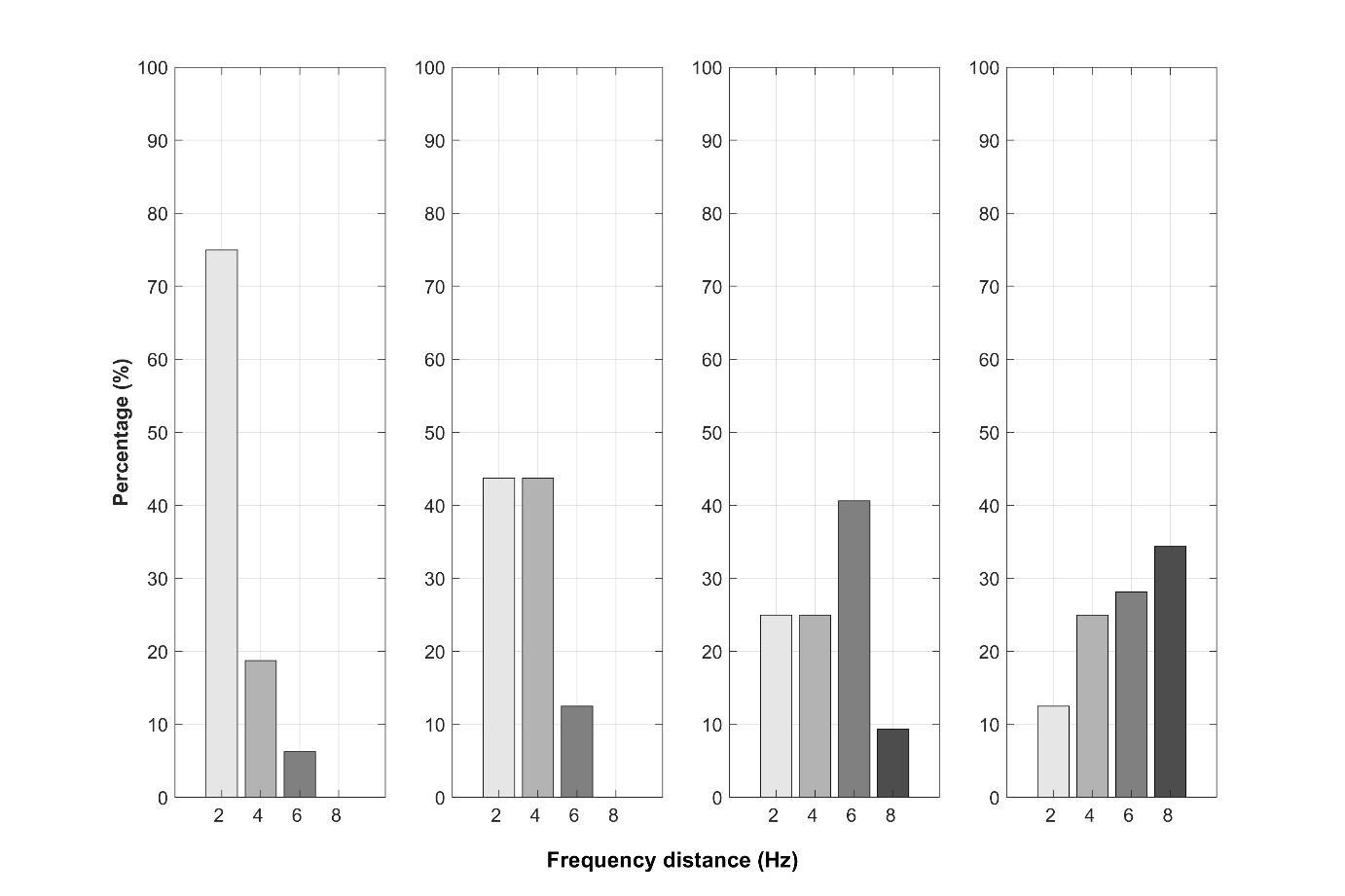


**Supplementary figure 1. Proportion of frequency distance between CF and NCF1 to NCF4**

Each figure represents the proportion of frequency distance between CF to NCF1, NCF2, NCF3, NCF4 (left to right).

Center frequency, CF; non-center frequency, NCF


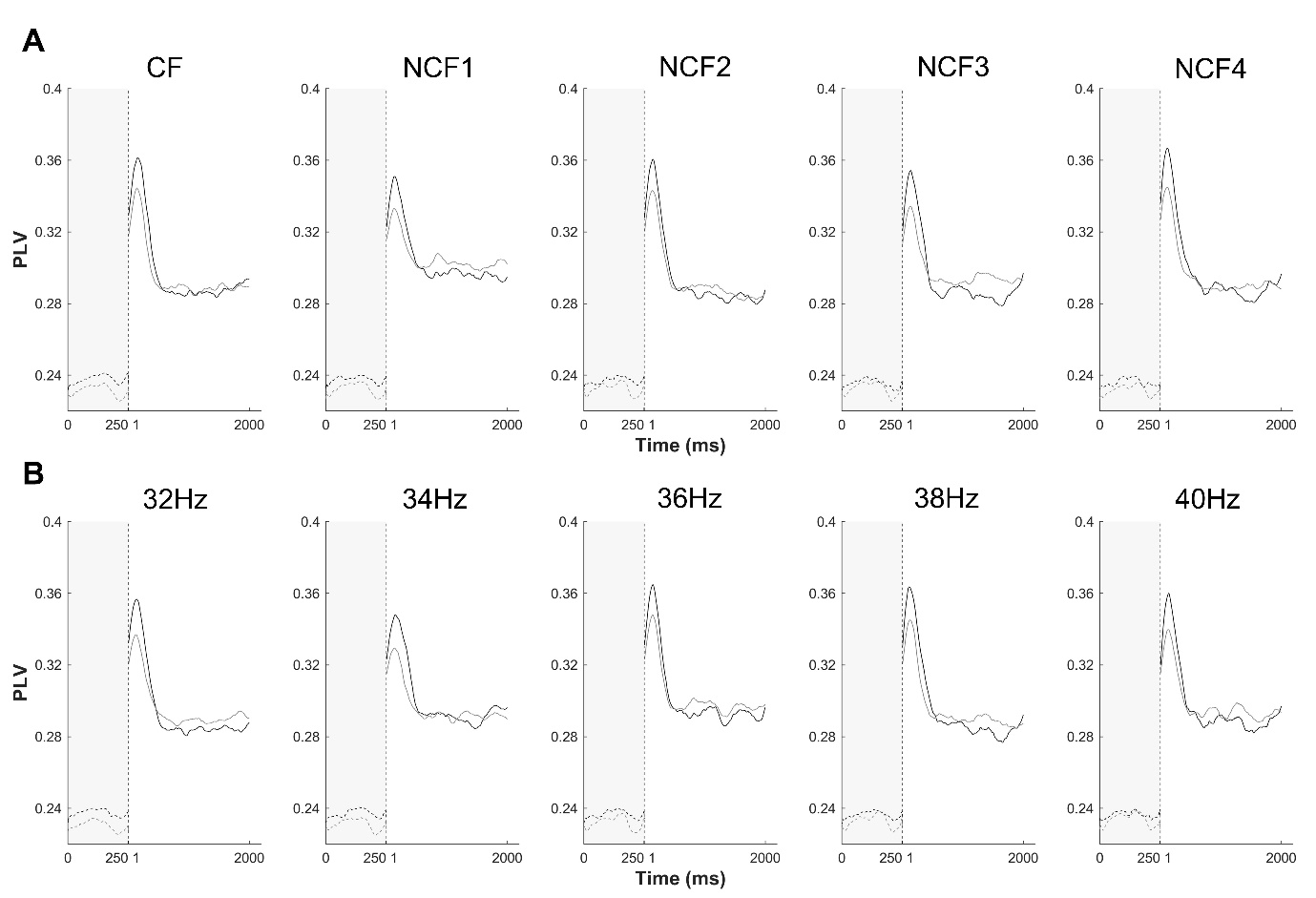


**Supplementary figure 2. Phase locking value over time across CF-NCFs and specific frequencies**

(A) shows PLV dynamics across various stimulus conditions, including CF and NCF1, NCF2, NCF3, and NCF4. (B) presents PLV changes for different stimulation frequencies, specifically 32 Hz, 34 Hz, 36 Hz, 38 Hz, and 40 Hz. In both panels, the shaded gray region represents 250ms of the resting-state PLV values, while the time range from 1 ms to 2000 ms depicts the PLV values recorded during FLS. The black lines represent the PLV of GC_V-NV_, while the gray lines represent the PLV of GC_NV-NV_.

Center frequency, CF; flickering light stimulation, FLS; non-center frequency, NCF; Phase Locking Value, PLV; gamma connectivity involving the visual cortex, GC_V-NV_; gamma connectivity that do not involve the visual cortex GC_NV-NV_.


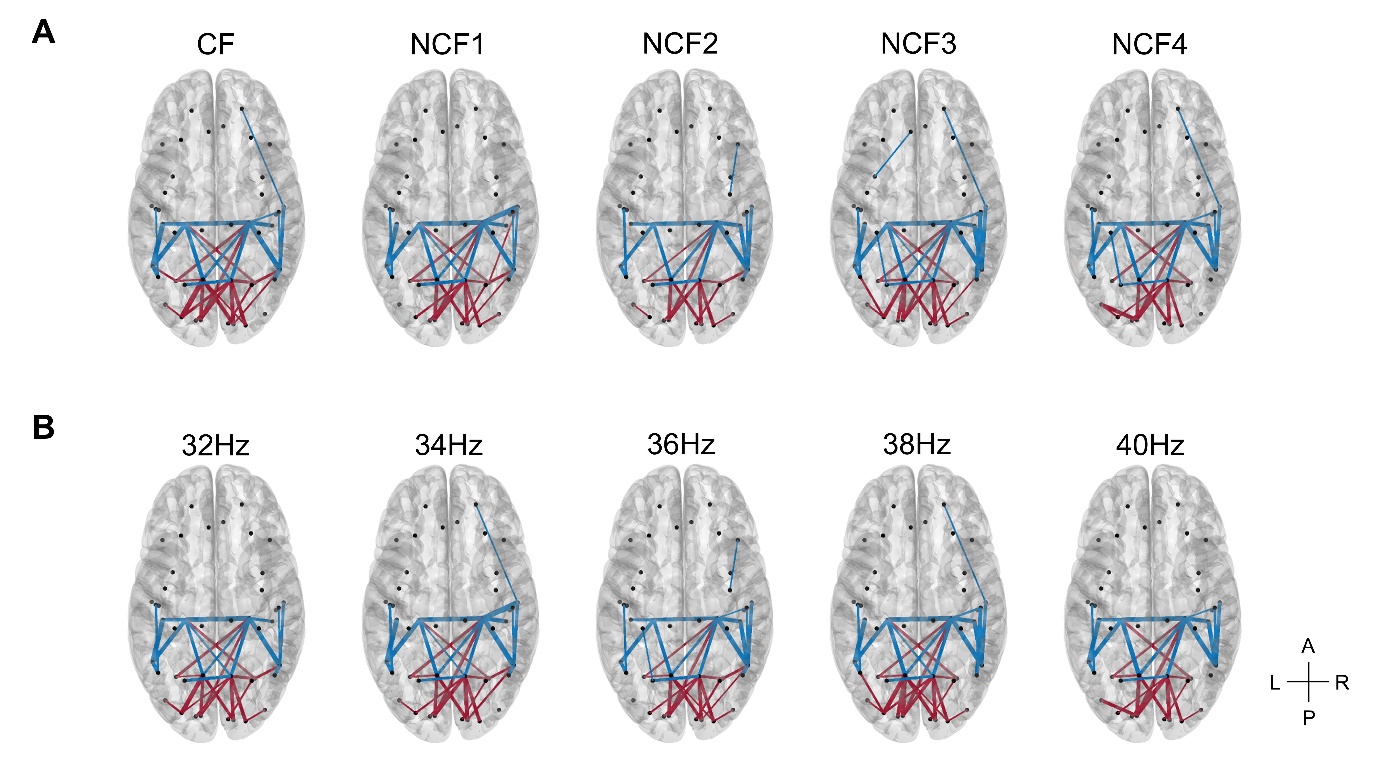


**Supplementary figure 3. Phase locking value-based connectivity in 3D brain source regions**

(A) Displays the PLV-based connectivity patterns across different stimulus conditions, including CF and NCF1, NCF2, NCF3, and NCF4. (B) Demonstrates the PLV connectivity across various stimulation frequencies: 32 Hz, 34 Hz, 36 Hz, 38 Hz, and 40 Hz. The thickness of the lines reflects the strength of the PLV. A strong connectivity threshold of 0.7 was applied to highlight robust connections. Red lines represent the connectivity between visual and non-visual regions, while blue lines depict the connectivity among non-visual regions.

Anterior, A; posterior, P; left, L; right, R; center frequency, CF; FLS: flickering light stimulation; non-center frequency, NCF; Phase Locking Value, PLV;
